# Spatiotemporal Analysis of the Primary Factors and Risk Quantification of Human Brucellosis Prevention and Control in Zhejiang Province of China

**DOI:** 10.1111/zph.70076

**Published:** 2026-07-22

**Authors:** Juan Li, Wenjing Li, Lingyan Zhao, Wei Jiang, Yongzhong Song, Jimin Sun, Hongli Zhang, Huaiping Zhu

**Affiliations:** ^1^ School of Computer Science and Technology (School of Artificial Intelligence) Zhejiang Sci‐Tech University Hangzhou People's Republic of China; ^2^ Ministry of Education Key Laboratory of NSLSCS, School of Mathematical Sciences Nanjing Normal University Nanjing People's Republic of China; ^3^ Zhejiang Provincial Center for Animal Disease Control and Prevention Hangzhou People's Republic of China; ^4^ Zhejiang Provincial Center for Disease Control and Prevention Hangzhou People's Republic of China; ^5^ LAMPS and Centre of Diseases Modeling CDM, Department of Mathematics and Statistics York University Toronto Canada

**Keywords:** Bayesian spatiotemporal model, decision support, prevention strategies, relative risk, risk factors, spatiotemporal analysis

## Abstract

**Introduction:**

Brucellosis is a major zoonotic disease that poses significant public health and socio‐economic threats. In China, human brucellosis incidence has continued to rise, including in highly developed regions such as Zhejiang Province, despite relatively low levels of livestock husbandry. This study investigates the recent surge in human brucellosis in Zhejiang, aiming to identify key risk factors and inform prevention strategies.

**Methods:**

We collected case data from 11 municipal divisions of Zhejiang Province (2018–2023). To overcome limitations of conventional clustering methods, we applied Bayesian spatiotemporal models, global autocorrelation analysis, variance inflation factor analysis and Spearman correlation analysis. Sheep and cattle production, socio‐economic metrics and climatic variables were incorporated to examine disease distribution, quantify risk factors and identify high‐risk areas.

**Results:**

Human brucellosis showed marked regional variation and spatiotemporal clustering. Elevated risks were observed in Huzhou, Jiaxing, Quzhou and Lishui, while Zhoushan, Ningbo, Taizhou, Wenzhou and Hangzhou displayed lower risks. Live sheep imports and slaughter volumes were the dominant factors, positively correlated with incidence (RR = 1.1816, 95% CI: 1.0096–1.3880; RR = 1.6175, 95% CI: 1.1883–2.3546). These findings underscore the importance of livestock trade and slaughter practices in shaping transmission dynamics.

**Conclusions:**

Targeted prevention strategies are urgently needed in developed regions such as Zhejiang. We recommend strengthening the brucellosis prevention framework, enhancing regulatory oversight of live sheep transport and slaughter in high‐risk areas and leveraging modern information technologies (e.g., AI and big data) for surveillance and decision support. This study advances the theoretical and practical framework for brucellosis prevention and offers insights for both regional and national disease control, contributing to public health protection and sustainable livestock development.

## Impacts

- Human brucellosis is rising even in wealthy, urbanized Zhejiang Province, China. Developed regions with limited local farming are still vulnerable, urging public health authorities everywhere to take this often‐overlooked disease more seriously.
- We found that live sheep imports and slaughter activities are the strongest predictors of human cases. Regulating livestock transport and improving hygiene at slaughterhouses are the most direct ways to reduce transmission risk.
- The study maps specific high‐risk cities and calls for AI and big data technologies to strengthen real‐time disease surveillance. These practical recommendations provide a replicable model for brucellosis control, applicable across China and other regions facing similar challenges.

## 1 Introduction

Brucellosis (also undulant fever, Mediterranean fever, Malta fever) is a zoonotic infection caused by *Brucella*, prevalent in localized regions worldwide (Cutler et al. 2005; Głowacka et al. 2018). It infects humans and over 60 animal species, with prominent cross‐species transmission (Shi and Lin 2014). In domestic animals, it causes reproductive disorders (e.g., abortion, mastitis in females; organ lesions in males) (Tian 2012). Human symptoms include arthralgia, persistent fever, hepatosplenomegaly and severe complications like infertility (Ma and Xue 2014). Globally, affecting 82.3% (144/175) of surveyed countries face human brucellosis threaths, with multi‐model evidence placing annual new cases at 1.6‐2.1million, far higher than the historic estimate of 500,000 (Laine et al. 2023; Beauvais et al. 2016; Eales et al. 2010), it threatens public health, impairs animal husbandry and disrupts livestock trade, worsening poverty (Franco et al. 2007; Kakkar et al. 2019). Brucellosis causes global direct economic losses of 1 ~ 3 billion USD yearly (Sheng 2014; Wang and Liu 2013). Given its severe impact and widespread dissemination, brucellosis is designated as a notifiable animal disease by the World Organization for Animal Health (WOAH) and classified as a Class II animal disease and a Class B infectious disease in China (Khurana et al. 2021). This emphasizes the critical need to address this disease on a global and national scale.

**FIGURE 1:**
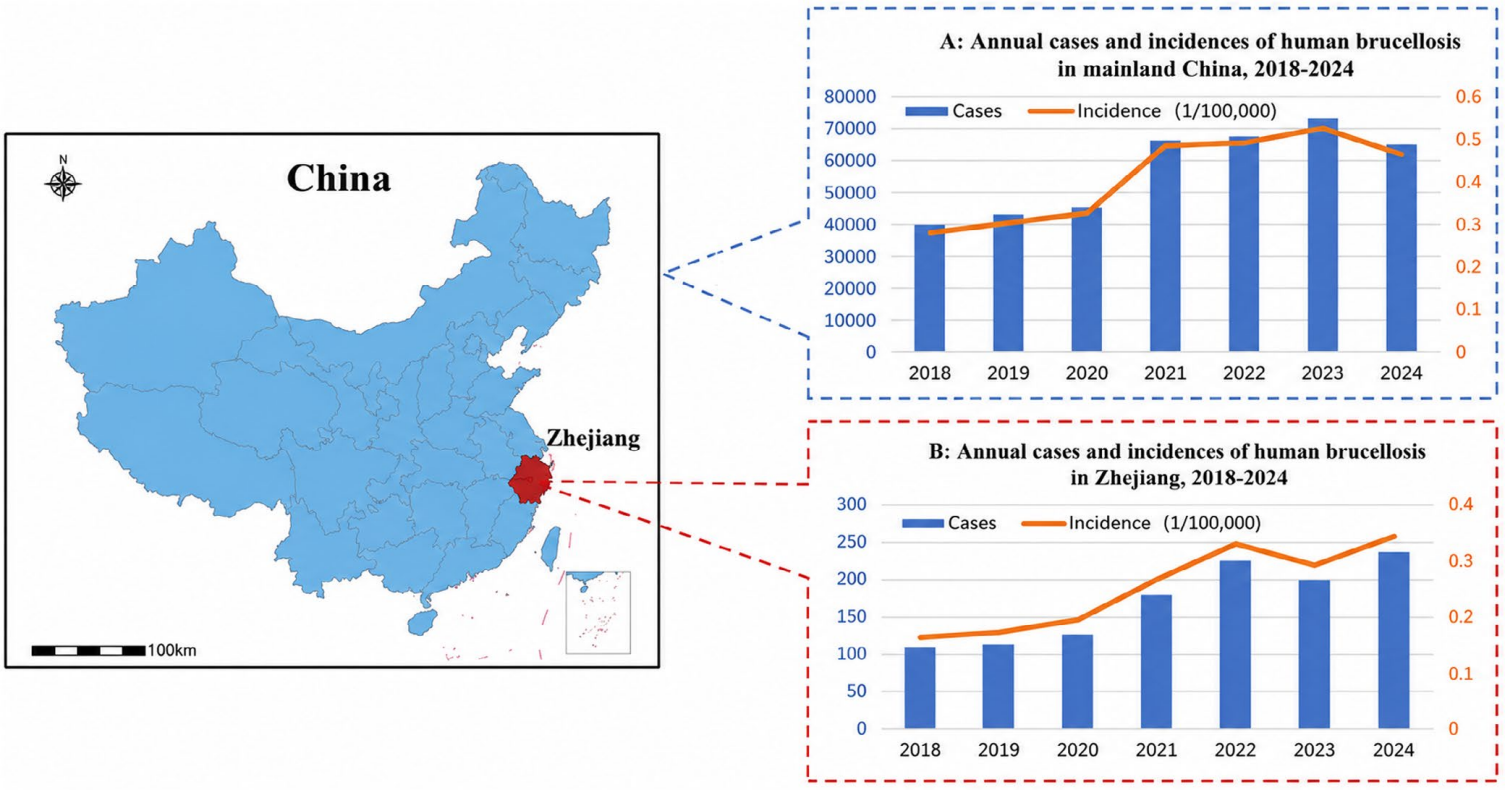
Number of human brucellosis cases and incidences in mainland China and Zhejiang (2018–2024). Data sources: Human brucellosis reported cases from the National Disease Control and Prevention Administration (NDCPA 2025) and Health Commission of Zhejiang Province (HCZP 2025); Resident population data from the National Bureau of Statistics of China (NBS 2024; 2025) and Zhejiang Provincial Bureau of Statistics (ZPBS 2024).

Despite national brucellosis prevention and control measures, China has seen a worsening epidemic in recent years: human cases surged from 37,911 (2.73/100,000) in 2018 to a peak of 75,858 (5.38/100,000) in 2023 (Figure 1A), highlighting gaps in current interventions that demand framework optimization. Aligning with the national ‘northward decline, southward rise’ trend (Zhang et al. 2024), southern regions have gained increasing attention. Zhejiang Province, located on China's southeast coast (27°02′‐31°11′ N, 118°01′‐123°10′ E), has encountered brucellosis since the 1950s linked to exotic cattle imports, with the disease spreading to 10 municipalities. Classified as a Class II area under China's ‘National Brucellosis Control Plan (2016–2020)’ (MARAPRC 2016), the province implemented monitoring and purification measures. However, challenges including widespread infection sources, funding shortages and weak grassroots epidemic prevention systems have hindered effectiveness. Zhejiang's human brucellosis cases have continued to rise, reaching a historical high of 239 reported cases (0.36/100,000) in 2024 (Figure 1B), exceeding prior‐year figures and posing an elevated public health challenge.

China's Ministry of Agriculture and Rural Development (MOA) has issued the ‘National Plan for Prevention and Control of Inter‐Animal and Zoonotic Diseases (2022–2030)’, designating brucellosis as a key disease and setting 2030 control targets (GPRC 2022). Concurrently, the ‘Five‐Year Action Program for Animal Brucellosis (2022–2026)’ concentrates on nine specific tasks (e.g., monitoring, immunization, disinfection, purification, supervision) to substantially reduce animal brucellosis prevalence and improve cattle/sheep health within 5 years (MARAPRC 2022). The Ministry of Agriculture and Rural Affairs Zhejiang Province has localized these policies via the ‘Five‐Year Action Program for Brucellosis Prevention and Control in Zhejiang Province (2022–2026)’ (ARAZP 2022). Nevertheless, continual monitoring and rigorous scientific assessment of these strategies are imperative to ensure their efficacy and appropriateness.

**FIGURE 2:**
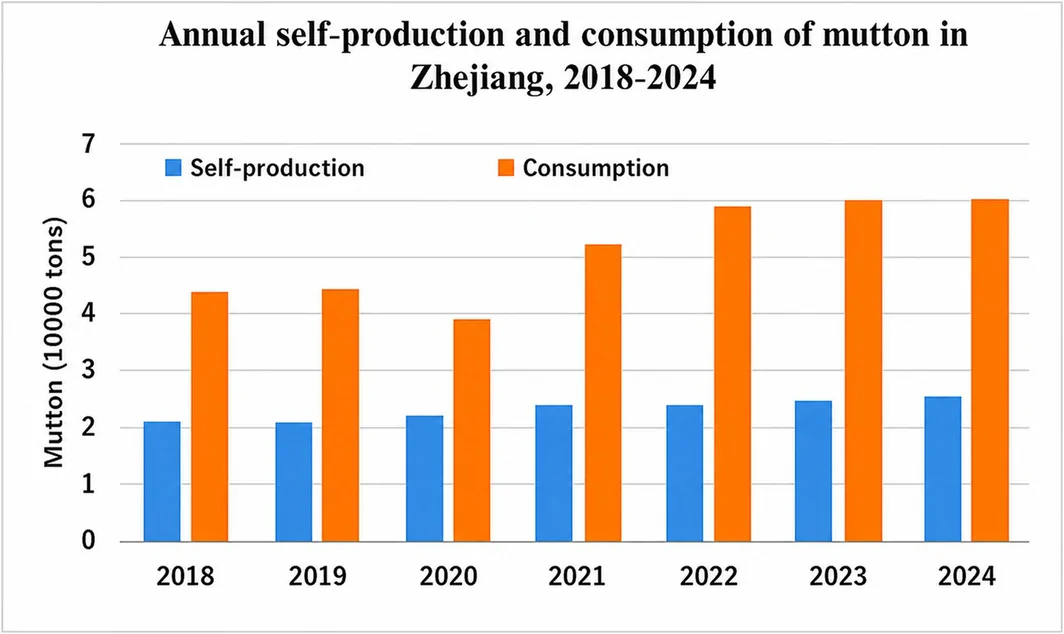
Annual self‐production and consumption of mutton in Zhejiang, 2018–2024, where annual self‐produced mutton ≈ annual slaughter × average mutton yield per sheep; annual consumption ≈ annual resident population × per capita mutton consumption. Data sources: Annual slaughter and per capita consumption of mutton from China Statistical Yearbook of National Bureau of Statistics of China (NBS 2024; 2025) and Survey Office of the National Bureau of Statistics in Zhejiang (SONBSZ 2024), average mutton production per sheep from China Animal Husbandry and Veterinary Medicine Yearbook (CSD 2024) and annual resident population statistics from Zhejiang Provincial Bureau of Statistics (ZPBS 2024).

Zhejiang Province, with complex epidemic dynamics and distinct geographic, economic and social contexts, serves as a key case for national brucellosis prevention and control. Covering ~105,500 km^2^ (11 municipal administrative units), the province had a 2023 population of over 66.27 million (high density) but limited land resources, resulting in inadequate local cattle/sheep self‐sufficiency (2023 slaughter: 1.3498 million sheep, 95,400 cattle). This necessitates substantial imports of live sheep (primarily from high‐risk regions), elevating the risk of introduced outbreaks. With economic and social development, Zhejiang's demand for beef, mutton and dairy products has surged (Figure 2), creating a market supply–demand gap that forces the livestock sector to rely on external sources, complicating brucellosis control. Additionally, rural revitalization‐driven expansion of animal husbandry has intensified livestock and product circulation, expanding transmission pathways. Brucellosis threatens local cattle/sheep farming, public health and biosecurity, with its transmission influenced by spatiotemporal, natural and social factors, presenting notable complexity.

Numerous studies have explored the spatiotemporal characteristics and influencing factors of brucellosis in China and specific provinces (Zhang et al. 2024; Liang et al. 2021; Wen et al. 2024; Peng et al. 2020; Sun et al. 2023; Liu et al. 2025; Yu et al. 2023), but their applicability to Zhejiang Province is constrained by the disease's complex transmission mechanisms shaped by temporal, spatial, natural and social factors. Relevant studies on Zhejiang have provided valuable insights: Liu et al. (2019) identified high‐risk occupational groups (sheep farming, slaughtering, transportation, processing) through 2018 case tracing; Wu et al. (2020) analysed 2010–2018 data, revealing an upward trend, spatial aggregation and ‘annual sheep slaughter’ as a key driver; Huang et al. (2024) noted a decline in sheep brucellosis (2018–2022) but highlighted severe situations in intensive farming/trading areas, emphasizing regulatory needs. However, current research primarily focuses on spatial clustering and basic statistics, neglecting dynamic spatiotemporal changes and interactive effects. Additionally, natural and social drivers of brucellosis spread are often studied in isolation, hindering the identification of underlying mechanisms. Thus, future research should establish an integrated dynamic analytical framework incorporating spatiotemporal dynamics and multifactorial interactions to quantify dominant factors and risks, elucidate epidemic root causes and support precise, science‐based brucellosis prevention and control in Zhejiang.

Bayesian spatiotemporal modelling is a robust tool for analysing complex multidimensional spatiotemporal data in infectious disease transmission, integrating Bayesian statistics' adaptability and spatiotemporal modelling precision to capture disease dynamics and accommodate data heterogeneity (Wang et al. 2024). Widely applied in spatial epidemiology, it has unravelled transmission patterns, spatiotemporal distributions and interacting drivers of diseases like malaria (Battle et al. 2019), hand‐foot‐mouth disease (He et al. 2020), schistosomiasis (Zhao et al. 2021), liver fluke (Xiao et al. 2023) and echinococcosis (Liu et al. 2022), informing prevention strategies and public health policies.

Targeting Zhejiang's brucellosis control needs, this study uses 2018–2023 municipal human brucellosis data, integrating multidimensional data (cattle/sheep production, population, economy, climate). Via Bayesian spatiotemporal modelling, it conducts descriptive statistics and municipal‐level analysis to explore the spatiotemporal distribution and trends of brucellosis relative risk (RR), quantify dominant transmission drivers and their impacts and evaluate regional risk levels. Results aim to enhance Zhejiang's brucellosis control precision and efficiency, optimise measure pertinence and provide insights for similar regions.

## 2 Materials and Methods

### 2.1 Dataset

Human brucellosis cases were reported according to the national diagnostic criteria (WS 269‐2019), classifying cases as suspected, clinically diagnosed, or laboratory confirmed based on epidemiological history, clinical presentation and serological or bacteriological evidence. The place of each case refers to the patient's registered residence (prefecture‐level city) in the National Infectious Disease Reporting System (NIDRS) and the date refers to the disease onset date. No major changes to the surveillance system or reporting criteria occurred during the 2018–2023 study period. All incidence rates reported are crude rates (cases per 100,000 resident population); age/sex‐standardized rates could not be calculated due to the absence of individual‐level demographic data in the aggregate dataset, which is acknowledged as a limitation. To comprehend the multitude of complex factors influencing the spread of brucellosis in Zhejiang Province, China, drawing insights from the analysis of factors affecting brucellosis in Inner Mongolia, China (Liang et al. 2021), this study systematically gathered and structured annual data from 11 prefecture‐level cities in Zhejiang Province spanning the years 2018 to 2023, encompassing 17 indicators. These indicators include direct feedback on brucellosis epidemiological status (number of human brucellosis cases), population structure (resident population size), economic development (Gross Domestic Product [GDP]) per capita, total regional GDP and per capita consumption expenditure), pastoral industry status (inventory, slaughter numbers, total yield of beef and mutton, imports of live cattle and sheep and by products volume of cattle and sheep), public health resource allocation (number of veterinary officers) and natural environmental parameters (average temperature and precipitation; included because environmental temperature and precipitation may influence Brucella survival in the environment, seasonal livestock movement patterns and occupational human‐animal contact—mechanisms documented in prior Chinese brucellosis studies (Peng et al. 2020; Sun et al. 2023; Wen et al. 2024).

Data sources are as follows: (1) Information on human brucellosis cases at the city level was provided by Zhejiang Provincial Center for Disease Control and Prevention (ZCDC); (2) Livestock and by‐product transfers and veterinary staff numbers were collected by Zhejiang Provincial Center for Animal Disease Control and Prevention (ZCADC); (3) Livestock inventory, slaughter numbers and meat production data were obtained from the Survey Office of the National Bureau of Statistics in Zhejiang (SONBSZ 2025); (4) Population and economic indicators (resident population, GDP per capita and total regional GDP) were extracted from official records documented in the Statistical Yearbook of Zhejiang Province (Li 2018; Shen 2023); (5) Climate data (average temperature and precipitation) were derived from the national historical temperature database of the 24 Network of Weather, calculated as annual averages over the study duration (24NW 2024). To ensure numerical stability, model robustness and convergence speed, all data underwent standardisation using the equation:

(1) $$x_{\text{scale}}=\frac{x-\mu }{S}$$

where $x_{\text{scale}}$ is the standardized value, x presents the original value, μ denoted the mean of x and S signifies the standard deviation of x.

### 2.2 Study Design

#### 2.2.1 Bayesian Spatiotemporal Models and Solution Methods

A Bayesian spatiotemporal model, grounded in Bayesian statistics, integrates spatial–temporal components to address heterogeneity and correlation in disease patterns by assuming observed variables (e.g., disease cases) follow specific distributions (Wang et al. 2024). Given the low annual number of newly diagnosed brucellosis cases in Zhejiang's cities, new cases ($Y_{\mathrm{it}}$) in the i‐th (1 i=1 ,2,...,N, N=11) region at time t‐th (t=1 ,2,...,T, T=6) are assumed to follows a Poisson distribution:

$$Y_{it}\sim \text{Poisson}\left(E_{it}\theta_{it}\right)$$

where $\theta_{\mathrm{it}}$ and $E_{it}$ denote the RR and the expected number of cases in the i‐th municipality at the t‐th time point, respectively. $E_{it}$ is calculated as expressed by:

(2) $$E_{\mathrm{ij}}=n_{\mathrm{ij}}*\left(\frac{\sum_{i=1}^{N}Y_{\mathrm{ij}}}{\Sigma_{i=1}^{N}n_{\mathrm{ij}}}\right),\left(n_{\mathrm{ij}}:\text{year}-\mathrm{end}\;\text{resident population}\right)$$

The model's general form (Kim et al. 2023) is

(3) $$\log\left(\theta_{\mathrm{it}}\right)=\beta_{0}+u_{i}+v_{i}+\varphi_{t}+\gamma_{t}+\delta_{\mathrm{it}}+\sum \beta_{k}X_{\mathrm{itk}}$$

where $\beta_{0}$ serves as the intercept term to quantify the average RR across all study areas. $u_{i}$ represents the spatial structure effect to characterise by the correlation across spatial area. Its prior distribution follows a Conditional Autoregressive Process (CAR) (Lee 2011). $v_{i}$ represents the spatial unstructured effect describing the heterogeneity within the region. $\varphi_{t}$ and $\gamma_{t}$ represent the temporal non‐structural and temporal structural effects, respectively; The prior distribution of the temporal non‐structural effect obeys the first‐order dynamic stochastic wandering model, Random Walk (1) (Xia et al. 2020). $\delta_{\mathrm{it}}$ represents the spatiotemporal interaction effect, describing the joint influence of temporal and spatial effects at different time points and locations. This effect varies across time and space, as detailed in Table 1 (Blangiardo et al. 2013a); $X_{\mathrm{itk}}$ denotes socioeconomic and meteorological factors associated with the RR, with $\beta_{k}$ as the regression coefficient. All hyperparameters except spatial–temporal structured effects follow $N\left(0,\sigma^{2}\right)$. The model was solved via INLA (Rue et al. 2009), a computationally efficient method for high‐dimensional sparse data with accurate approximations (Blangiardo et al. 2013b; Gelfand and Smith 1985; Illian et al. 2013).

**TABLE 1:** Spatiotemporal interaction term representation.

Form of expression ($\delta_{\mathrm{it}}$)	Interaction term
Type 1	$v_{i}\&\varphi_{t}$
Type 2	$v_{i}\&\gamma_{t}$
Type 3	$u_{i}\&\varphi_{t}$
Type 4	$u_{i}\&\gamma_{t}$

#### 2.2.2 Evaluation Criteria for Bayesian Spatiotemporal Models

Model quality was evaluated using two standard criteria: Deviance Information Criterion (DIC; Spiegelhalter et al. 2002) and Watanabe‐Akaike Information Criterion (WAIC; Watanabe 2010), both balancing fit and complexity with lower values indicating better performance. DIC is calculated as:

(4) $$\mathrm{DIC}=D\left(\bar{\theta }\right)+2p_{D}=\bar{D}+p_{D}$$

where $D\left(\bar{\theta }\right)$ denotes the deviation from the posterior mean and $\bar{D}$ represents the posterior mean of the deviation, serving as a measure of model fit. $p_{D}$ is the effective number of parameters (complexity). And WAIC is computed as:

(5) $$\text{WAIC}=-2\times l\mathrm{ppd}+2\times p_{\text{WAIC}}$$

with lppd (log predictive density) summing the log‐likelihood for each data point and $p_{\text{WAIC}}$ quantifying complexity via likelihood variance. WAIC offers more accurate generalisation error estimation and data point‐specific fit quantification than DIC but may exhibit greater bias in complex models or those with extensive missing data. Given their complementary strengths and limitations, both criteria were combined for comprehensive model assessment.

#### 2.2.3 Global Autocorrelation Analysis

Global autocorrelation analysis via Global Moran's I (Westerholt 2022) was performed to characterise the spatial distribution of brucellosis relative risk (RR, θ) derived from the Bayesian spatiotemporal model (here, X=θ) and crude incidence rate (CIR), quantifying spatial interdependence, aggregation degree and supporting model validity. The Moran index I is calculated as:

(6) $$I=\frac{N}{W}\frac{\Sigma_{i=1}^{N}\Sigma_{j=1}^{N}}{\Sigma_{i=1}^{N}}\frac{w_{ij}\left(X_{i}-\bar{X}\right)\left(X_{j}-\bar{X}\right)}{{\left(X_{i}-\bar{X}\right)}^{2}}$$

where $w_{ij}$ represents the spatial weight matrix, W is the sum of all spatial weights, that is, $W=\Sigma_{i=1}^{N}\Sigma_{j=1}^{N}w_{ij}$, $X_{i}$ and $X_{j}$ are the observations for regions i and j, respectively, $\bar{X}$ is the mean observation and *N* is the total observations. The interpretation of Moran Index I is as follows: I>0 indicates a positive correlation, I<0 indicates a negative correlation and I=0 indicates spatial random distribution. Significance was tested via standardised:

(7) $$Z\left(I\right)=\frac{1-E\left(I\right)}{\sqrt{\mathrm{Var}\left(I\right)}}$$

with p<0.05 indicating statistically significant spatial autocorrelation. This analysis described the annual spatial pattern of brucellosis RR and CIR in Zhejiang Province (2018–2023). The CIR‐based Moran's I was calculated to characterise the baseline spatial autocorrelation of brucellosis prior to Bayesian spatiotemporal modelling. The RR‐based Moran's I was retained as a post hoc model adequacy diagnostic to verify that the spatially structured conditional autoregressive (CAR) component had adequately absorbed residual spatial autocorrelation.

#### 2.2.4 Analysis of Influencing Factors

To identify key factors explaining brucellosis spread, multicollinearity was first addressed via Variance Inflation Factor (VIF; Chien et al. 2022), with variables having a condition index > 10 excluded. Univariate Bayesian spatiotemporal models were then used to assess incidence relative risk (RR), with variables showing 95% confidence intervals excluding ‘1’ deemed significant. These significant variables were further screened to minimise multicollinearity before inclusion in the multifactorial model, which quantified independent and combined effects of factors to support brucellosis factors driving incidence in Zhejiang Province. Spearman correlation analysis (Dormann et al. 2013) was applied to excluded variables, with | ρ| > 0.05 indicating potential collinearity.

### 2.3 Software

Data were organized in Microsoft Excel. R 4.2.3 was used for correlation analysis, Bayesian spatiotemporal model construction, spatiotemporal analysis, influencing factor evaluation, global spatial autocorrelation analysis of brucellosis RR and generation of spatiotemporal distribution maps for visualizing results.

## 3 Results

### 3.1 Descriptive Analysis

#### 3.1.1 Temporal Characteristics

**FIGURE 3:**
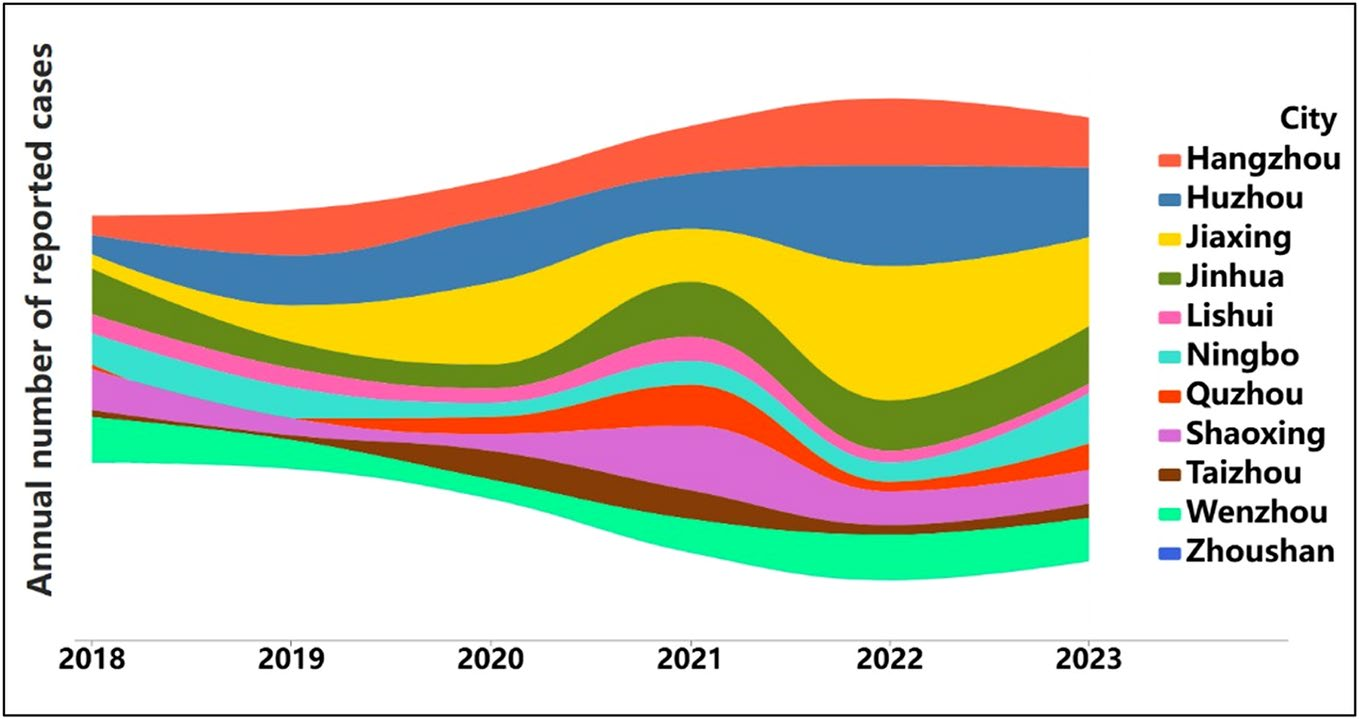
Annual reported cases of human brucellosis by city in Zhejiang, 2018–2023.

From 2018 to 2023, Zhejiang Province recorded 954 cumulative brucellosis cases, with annual reported cases ranging from 108 to 221. Figure 3 presents the temporal dynamics of annual human brucellosis cases across Zhejiang Province and its municipalities via a river diagram, where the width of each ‘river’ directly reflects the incidence scale of the region and year in question.

During the observation period, brucellosis cases in Zhejiang experienced an overall increasing trend with heterogeneous varying growth rates. As reflected in the river diagram: slow widening of rivers during 2018–2020 indicated stable disease spread; a sharp widening in 2020–2021 signalled accelerated transmission; and slight narrowing in 2022–2023 suggested a growth slowdown or reversal. Notable regional variations in incidence patterns were observed across municipalities: Huzhou and Jiaxing exhibited ‘increase–decrease–increase’ fluctuations, peaking in 2022, highlighting local prevention and control challenges. Wenzhou and Shaoxing followed a reverse ‘decrease–increase–decrease’ pattern, with peaks in 2022 and 2021 respectively, reflecting alternating effectiveness of control measures and recurrent outbreaks. Hangzhou and Taizhou showed ‘increase–decrease–increase’ trends: Hangzhou's post‐2022 river narrowing indicated successful control, while Taizhou maintained upward momentum. Quzhou and Jinhua experienced ‘decrease–increase–decrease‐again’ cycles before shifting upward, with the 2021 peak prompting strategic adjustments in regional control. Lishui and Ningbo initially showed stable trends but transitioned to ‘decrease–increase–decrease’ after 2019: Lishui continued declining, while Ningbo reversed course, with peaks in 2021 and 2023 respectively.

#### 3.1.2 Spatial Characteristics

**FIGURE 4:**
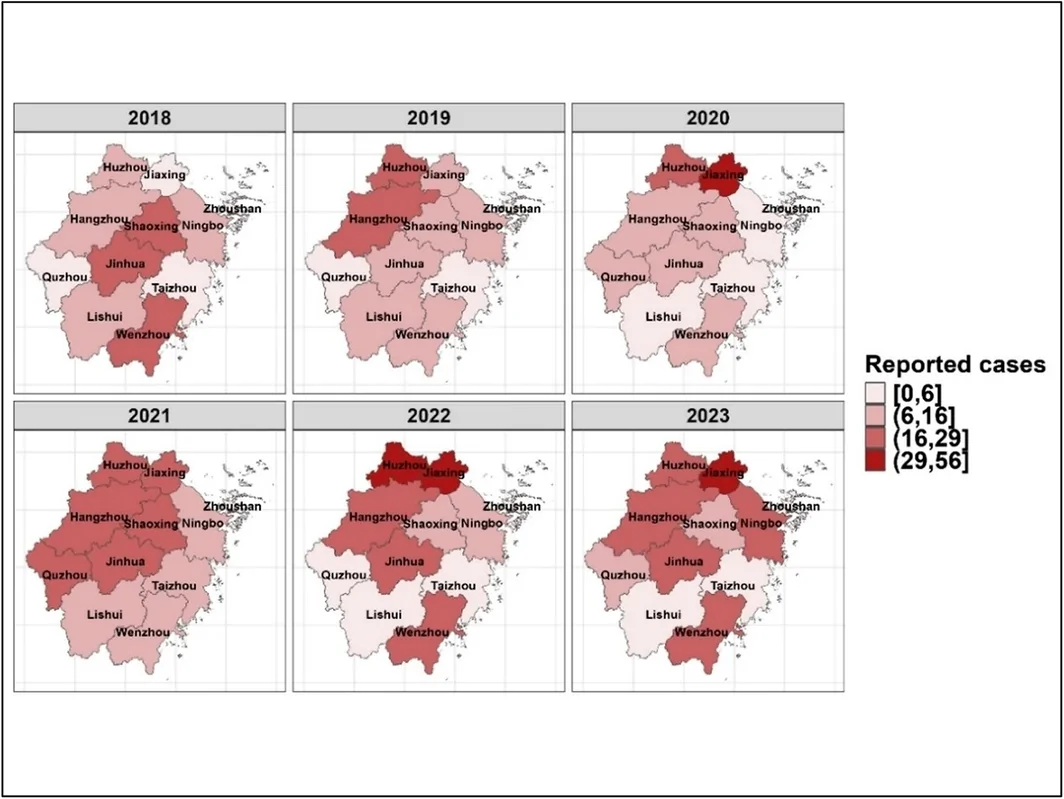
Spatial and temporal distribution of annual reported cases of human brucellosis at the city level in Zhejiang, 2018–2023.

Between 2018 and 2023, brucellosis cases were reported in 10 cities of Zhejiang, excluding Zhoushan. The spatial distribution of cumulative cases (visualized via the natural point break method (Ke et al. 2023) in Figure S2) reveals a significant clustering: high reported cases (110–170 cases) in northwestern Huzhou, Jiaxing and Hangzhou; moderate‐high (86–110 cases) in southern Wenzhou and central Jinhua; moderate (41–85 cases) in northeastern Shaoxing and Ningbo; and low (0–41 cases) in Quzhou, Lishui, Taizhou and Zhoushan. Furthermore, Figure 4 illustrates the annual regional case distribution. In 2018, Shaoxing, Jinhua and Wenzhou exhibited the highest infection loads (16–29 cases). The subsequent year saw Wenzhou and Hangzhou leading with comparable case ranges. In 2020, Jiaxing reported the highest number of cases (29–56 cases). By 2021, cases predominantly occurred in northwestern Zhejiang, particularly in Hangzhou, Huzhou and Quzhou. During 2022, Huzhou and Jiaxing again reported the highest case counts (29–56 cases), closely followed by Hangzhou, Jinhua and Wenzhou. In 2023, Jiaxing led with the highest reported cases (29–56 cases), followed by Huzhou, Hangzhou, Jinhua, Ningbo and Wenzhou (16–29 cases). These spatial patterns reflect geographic clustering of brucellosis, suggesting environmental or socio‐economic drivers of transmission. The northwest region (Huzhou, Jiaxing, Hangzhou) consistently emerged as a high‐risk zone, implying potential shared determinants such as disease vectors, livestock management practices, or human exposure pathways.

#### 3.1.3 Age and Sex Distribution

**FIGURE 5:**
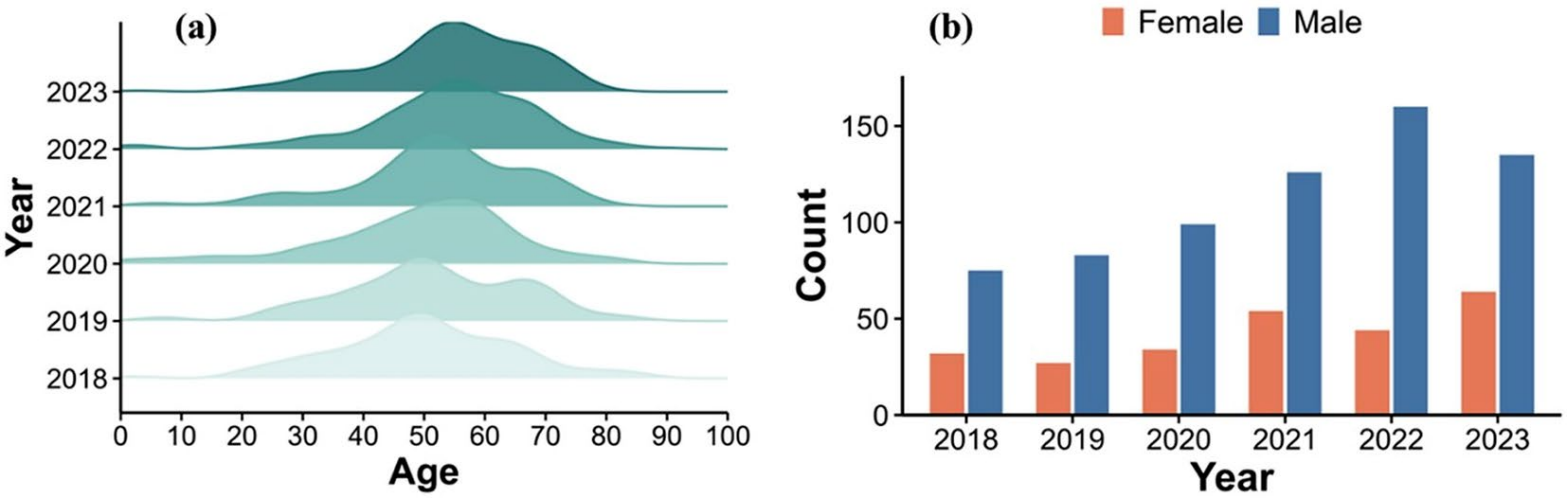
Sex and age distribution of the 954 reported cases in Zhejiang over the study period (2018–2023).

Regarding demographic characteristics, age distribution presented a unimodal pattern, with the 40–60 years age group as the peak incidence interval. This group accounted for 52.84% of cases with complete age data (Figure 5A), identifying them as the core high‐risk population for brucellosis in Zhejiang Province. Significant gender disparities were observed: males accounted for 72.67% of all cases, nearly three times the proportion of females, indicating a higher infection risk among males (Figure 5B).

#### 3.1.4 Occupational Exposures

**FIGURE 6:**
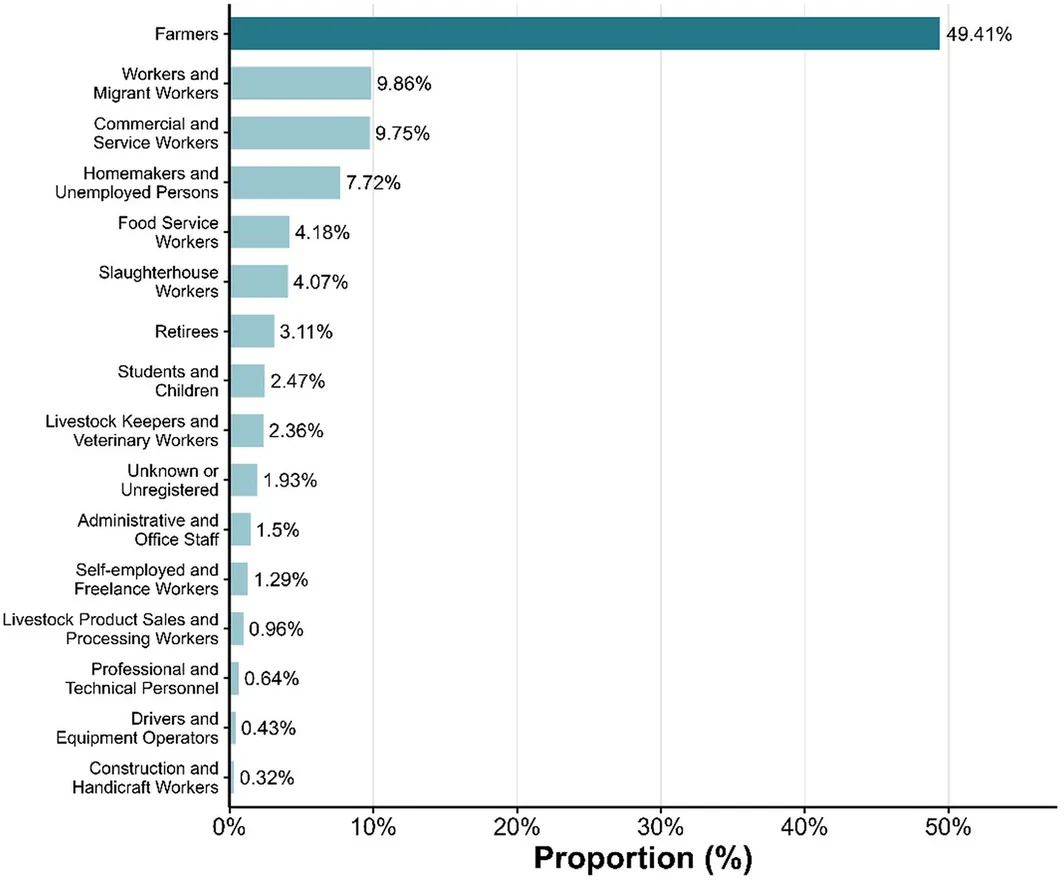
Occupational exposures of the 954 reported cases in Zhejiang over the study period (2018–2023).

As shown in Figure 6, the occupational distribution of brucellosis cases was dominated by farmers, who accounted for 49.41% of all cases. This was followed by workers/migrant workers (9.86%) and commercial service workers (9.75%, 91/933). Among groups with direct occupational exposure to brucellosis, slaughterhouse/sheep slaughter workers accounted for 4.07%, livestock keepers/veterinary workers for 2.36% and workers involved in the sale and processing of cattle and sheep products for 1.0%. When farmers, livestock keepers/veterinary workers, slaughterhouse/sheep slaughter workers and workers in cattle/sheep product sales and processing were further categorized into the ‘animal contact or animal product exposure‐related’ group, the results showed that 56.80% of cases had exposure backgrounds related to agricultural production, livestock farming, livestock slaughtering, or animal product processing. These findings indicate that reported brucellosis cases in Zhejiang Province remain concentrated in populations engaged in agricultural production and with direct contact with cattle and sheep, representing the core high‐risk groups for infection.

### 3.2 Spatiotemporal Analysis Based on Spatiotemporal Bayesian Model

Six Bayesian spatiotemporal models were fitted to analyse the influencing factors and spatiotemporal trends of brucellosis. Model A captures spatial heterogeneity in temporal trends through a random coefficient framework; Model B includes only independent spatial and temporal random effects without additional spatiotemporal interaction; and Models C–F adopt different specifications of structured or unstructured spatiotemporal interaction terms to describe how spatial effects evolve over time. Model selection was based on DIC and WAIC (lower values indicate better fit), with Model C exhibiting the minimum indices (Table 2). Consistent with the principle of minimizing these evaluation criteria, Model C was selected as the reference model for subsequent analysis.

**TABLE 2:** Fitting effects of six Bayesian spatiotemporal models.

Number	Models	Interaction type	DIC	WAIC
A	$\log\left(\theta_{\mathrm{it}}\right)=\beta_{0}+u_{i}+v_{i}+\left(\beta_{1}+\delta_{i}\right)*t_{j}$	Random coefficient interaction	422.8596	444.1912
B	$\log\left(\theta_{\mathrm{it}}\right)=\beta_{0}+u_{i}+v_{i}+\varphi_{t}+\gamma_{t}$	Independent space–time effects	422.91	444.87
C	$\log\left(\theta_{\mathrm{it}}\right)=\beta_{0}+u_{i}+v_{i}+\varphi_{t}+\gamma_{t}+\delta_{\mathrm{it}1}$	Type 1 in Table 1	370.85	368.95
D	$\log\left(\theta_{\mathrm{it}}\right)=\beta_{0}+u_{i}+v_{i}+\varphi_{t}+\gamma_{t}+\delta_{\mathrm{it}2}$	Type 2 in Table 1	373.02	384.52
E	$\log\left(\theta_{\mathrm{it}}\right)=\beta_{0}+u_{i}+v_{i}+\varphi_{t}+\gamma_{t}+\delta_{\mathrm{it}3}$	Type 3 in Table 1	380.43	391.01
F	$\log\left(\theta_{\mathrm{it}}\right)=\beta_{0}+u_{i}+v_{i}+\varphi_{t}+\gamma_{t}+\delta_{\mathrm{it}4}$	Type 4 in Table 1	372.23	382.82

#### 3.2.1 Spatial Distribution Analysis

**FIGURE 7:**
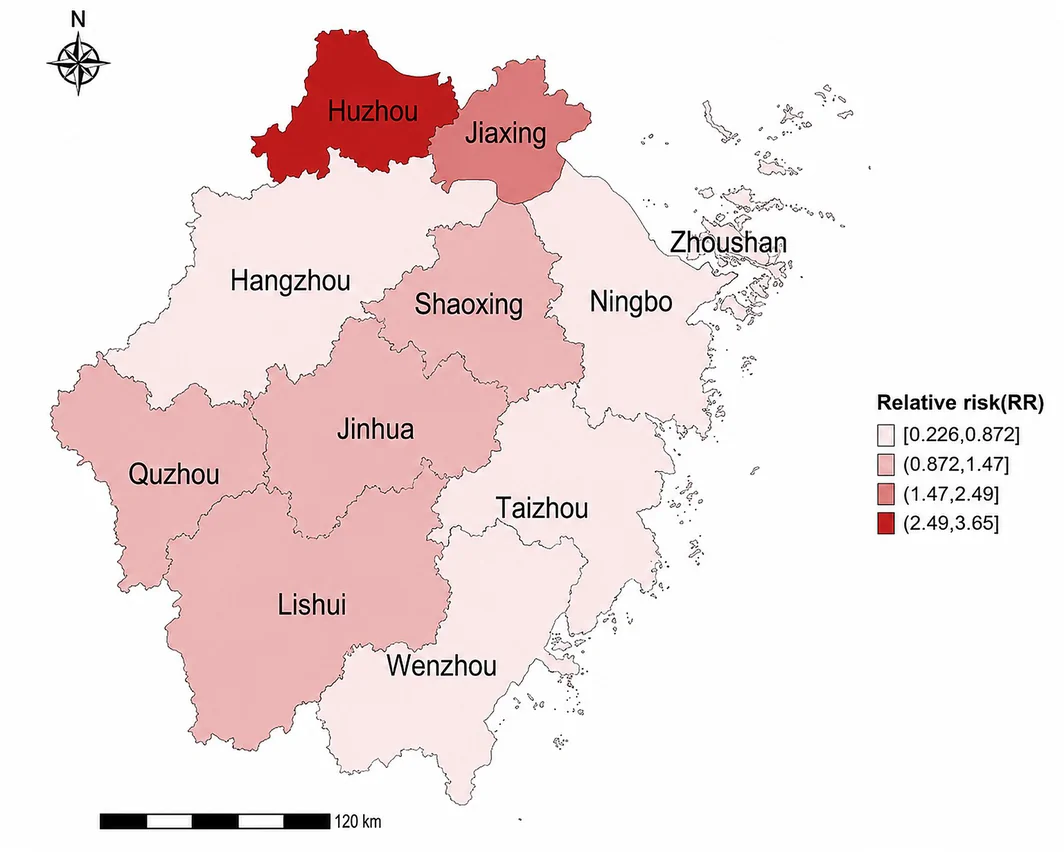
Spatial distribution of RR of human brucellosis in each region in Zhejiang.

The Bayesian spatiotemporal model was employed to calculate the RR mean via $e^{u_{i}+v_{i}}$, assessing the spatial effect of brucellosis incidence across Zhejiang's municipalities. Figure 7 presents the 2018–2023 spatial risk distribution (natural break method, four levels), showing distinct spatial aggregation: Huzhou (northwest) as severe high‐risk, adjacent Jiaxing as high‐risk, Shaoxing/Jinhua/Quzhou/Lishui as medium‐risk and Ningbo/Taizhou/Zhoushan/Wenzhou/Hangzhou (eastern regions) as low‐risk.

**FIGURE 8:**
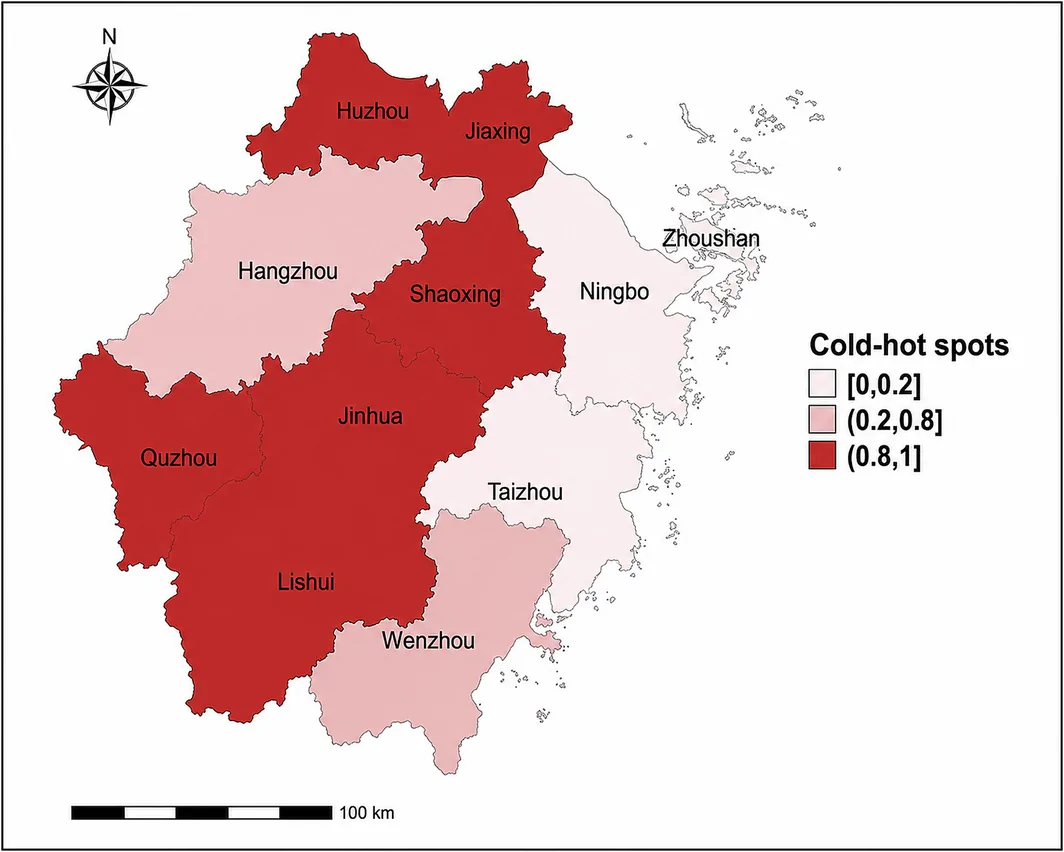
Cold‐hot spot analysis of RR of human brucellosis at the city level in Zhejiang, 2018–2023, where hot spots refer to municipalities with posterior probability (PP) ≥ 0.8, indicating an over 80% probability of elevated disease risk, while cold spots are defined as those with PP ≤ 0.2.

The posterior probability (PP) map (Figure 8) displays $P\left(\mathrm{RR}>1\;|\;\text{data}\right),$ that is, the probability that the relative risk in each municipality exceeds the provincial average given the observed data, derived from the INLA posterior distribution. Hot spots are defined as municipalities with PP ≥ 0.8 and cold spots as municipalities with PP ≤ 0.2. For further spatial pattern analysis, Figure 7 illustrates cold/hot spot distributions: six hot spots (Huzhou, Jiaxing, Shaoxing, Jinhua, Lishui, Quzhou) with significantly higher incidence risk than the provincial average (high posterior probabilities); Hangzhou and Wenzhou as non‐significant spots; and Zhoushan, Ningbo, Taizhou as cold spots with significantly lower risk (low posterior probabilities).

#### 3.2.2 Global Autocorrelation Analysis

Table 3 shows the Global Moran's I indices for the pre‐model CIR and model‐fitted RR of brucellosis in Zhejiang Province from 2018 to 2023. The RR values exhibited significant positive spatial autocorrelation in 2019 and 2021–2023 (*I* > 0, *p* < 0.05), whereas no significant spatial autocorrelation was observed in 2018 and 2020 (*p* > 0.05). For the pre‐model CIR, significant spatial autocorrelation was detected in 2020 and 2022 (*p* < 0.05), while no statistically significant spatial patterns were found in 2018, 2019, 2021 and 2023 (*p* > 0.05). These results confirm that the spatiotemporal model effectively captured the underlying spatial structure of brucellosis incidence.

**TABLE 3:** RR and CIR of human Brucellosis global Moran's I Index, Zhejiang, 2018–2023.

Year	RR		CIR	
	Moran's I	*p* value	Moran's I	*p* value
2018	−0.0769	0.4576	−0.2434	0.7562
2019	0.1839	0.0406	−0.0251	0.1795
2020	0.1229	0.1472	0.1734	0.0406
2021	0.2401	0.0231	−0.1270	0.5343
2022	0.3946	0.0040	0.2389	0.0137
2023	0.2846	0.0236	0.1174	0.0927

#### 3.2.3 Temporal Effect Analysis

**FIGURE 9:**
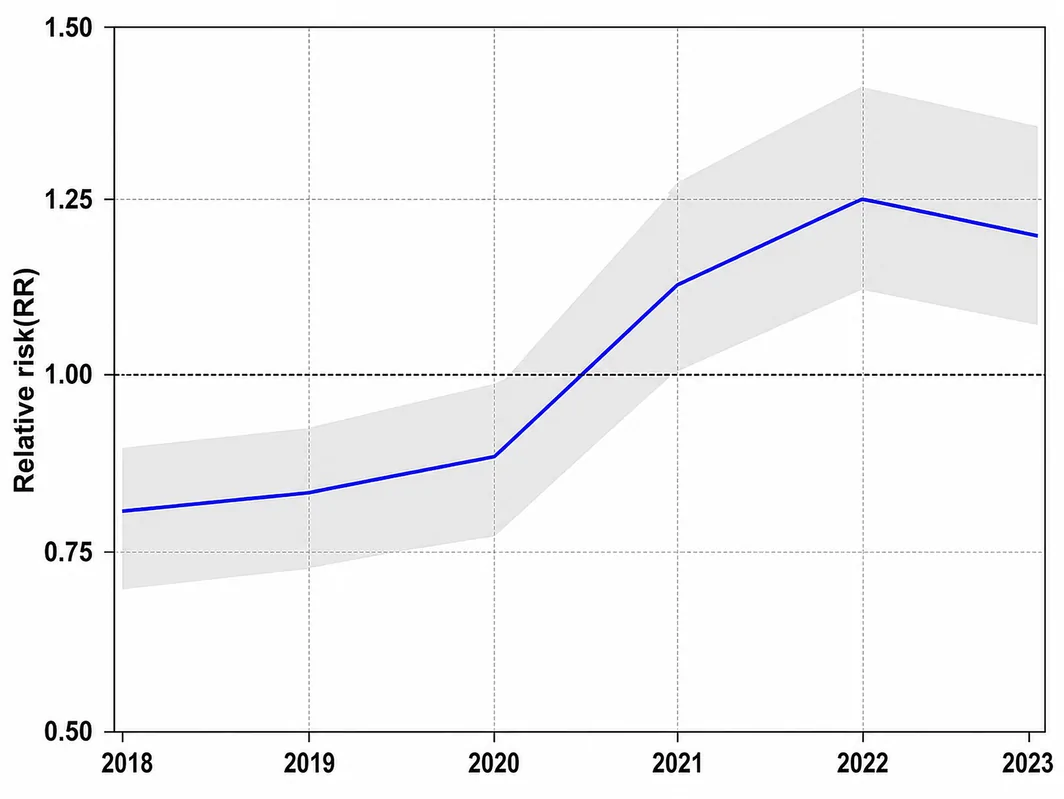
Temporal trend of RR of human brucellosis in Zhejiang from 2018 to 2023, shaded area: 95% credible interval of posterior mean RR.

**FIGURE 10:**
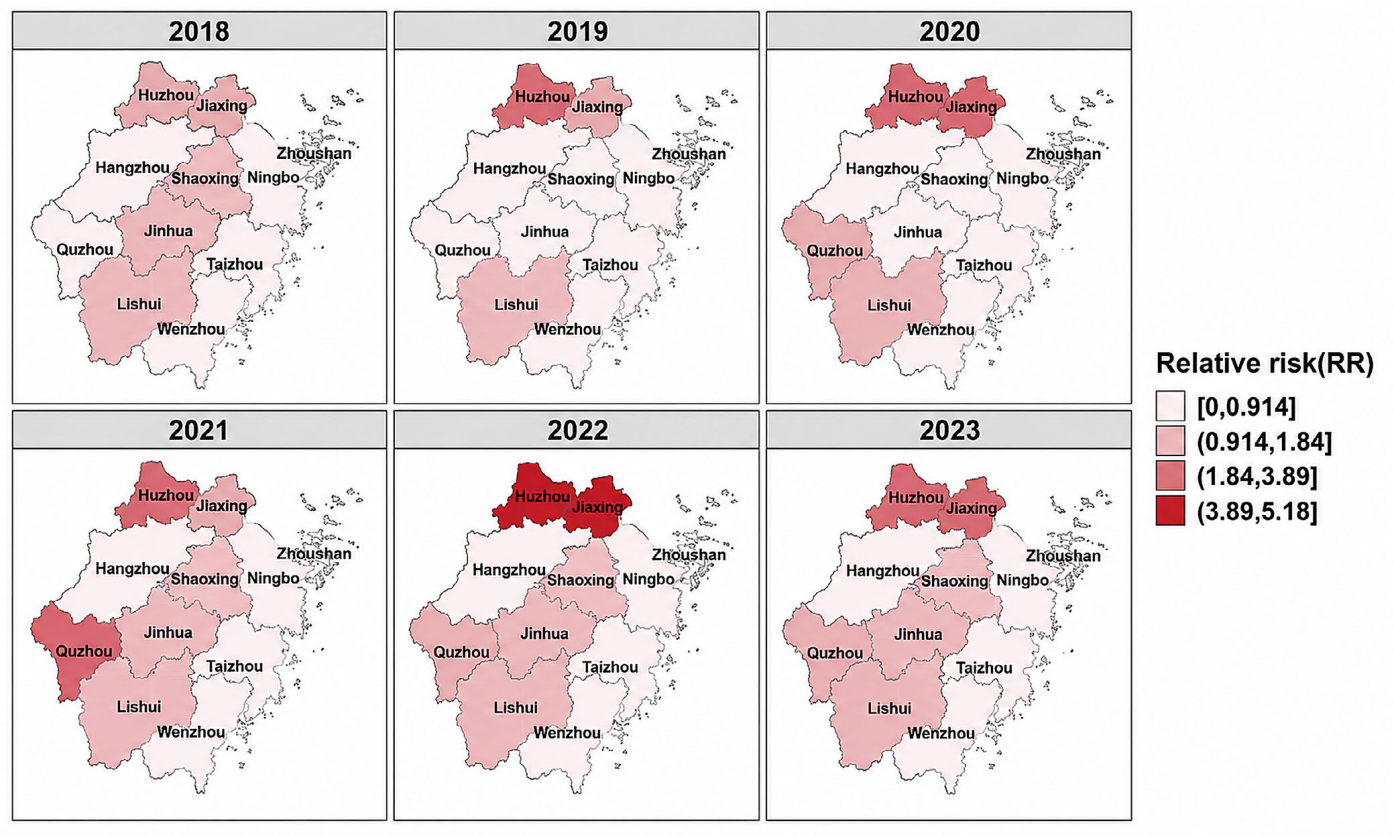
Temporal and spatial variation of RR of brucellosis in each region in Zhejiang Province, 2018–2023.

The Bayesian spatiotemporal model calculates the global temporal effect via $e^{\left(\varphi_{t}+\gamma_{t}\right)}$. Figure 9 illustrates the 2018–2023 brucellosis relative risk (RR) trend across Zhejiang's 11 municipalities, presenting an ‘increase‐then‐decrease’ pattern: overall RR remained low (< 1) before 2020, surged above 1 post‐2020 and aligned with Figure 10 trend. RR peaked in 2022 (high epidemic risk) and decreased in 2022–2023. This fluctuation underscores the dynamic nature of brucellosis and the necessity of continuous monitoring and adaptive public health strategies. The shaded area in Figure 8 represents the 95% credible interval of the posterior mean RR derived from the INLA posterior distribution.

#### 3.2.4 Analysis of Spatial and Temporal Distribution

To visualise the 2018–2023 monthly incidence rate and relative risk (RR) dynamics across Zhejiang's regions, a four‐tier risk classification (low/medium/high/severe high) was adopted using the natural breaking method (Figures 9 and 10). Figure 10 shows spatial clustering of RR: northern Huzhou shifted from high to severe high risk (2018–2022) and maintained elevated risk, with neighbouring Jiaxing following a similar upward trend (slightly lower RR); Shaoxing/Jinhua exhibited consistent trends, while Quzhou/Lishui (spatial hotspots) showed comparable RR changes. Lower risks concentrated in Zhoushan, Ningbo, Taizhou, Wenzhou and eastern Hangzhou. The provincial average RR rose from 0.895 in 2018 (5 high‐risk/6 low‐risk areas vs. provincial average) to 1.247 in 2023, indicating an overall upward risk trend. The year‐by‐year temporal evolution of the posterior probability $P\left(\mathrm{RR}>1\right)$ across Zhejiang's municipalities from 2018 to 2023 is further presented in the supplementary panel (Figure S1).

**FIGURE 11:**
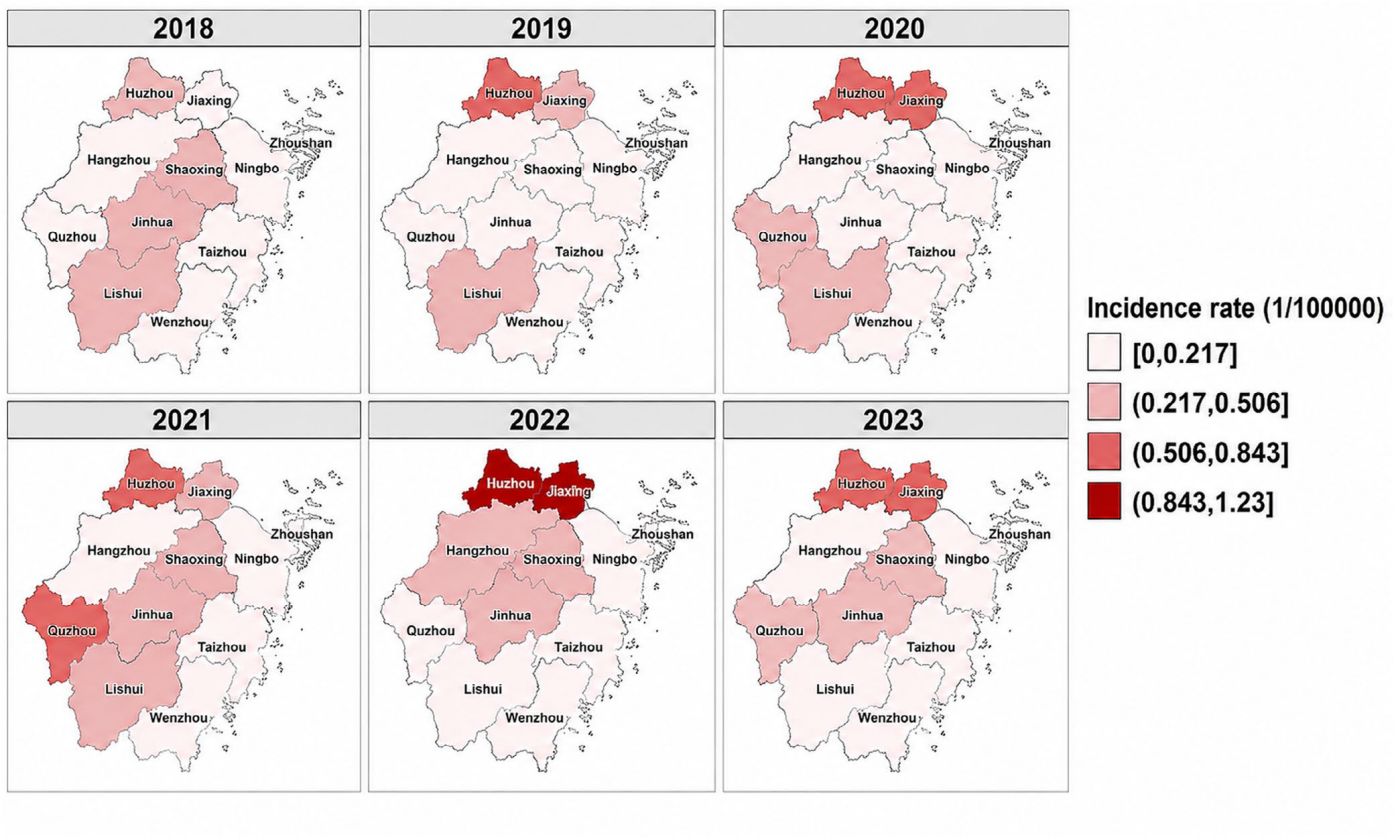
Incidence rate of brucellosis in each region in Zhejiang Province, 2018–2023.

Comparing Figures 10 and 11 highlights factor‐induced discrepancies in risk classification: for example, Jiaxing in 2018 and Lishui in 2023 were low‐risk by incidence rate but medium‐risk by RR; Hangzhou in 2022 was medium‐risk by incidence rate but low‐risk by RR; Quzhou and Lishui showed reversed classifications between the two metrics.

### 3.3 Analysis of the RR of the Influencing Factors

#### 3.3.1 Multi‐Collinearity Analysis

**FIGURE 12:**
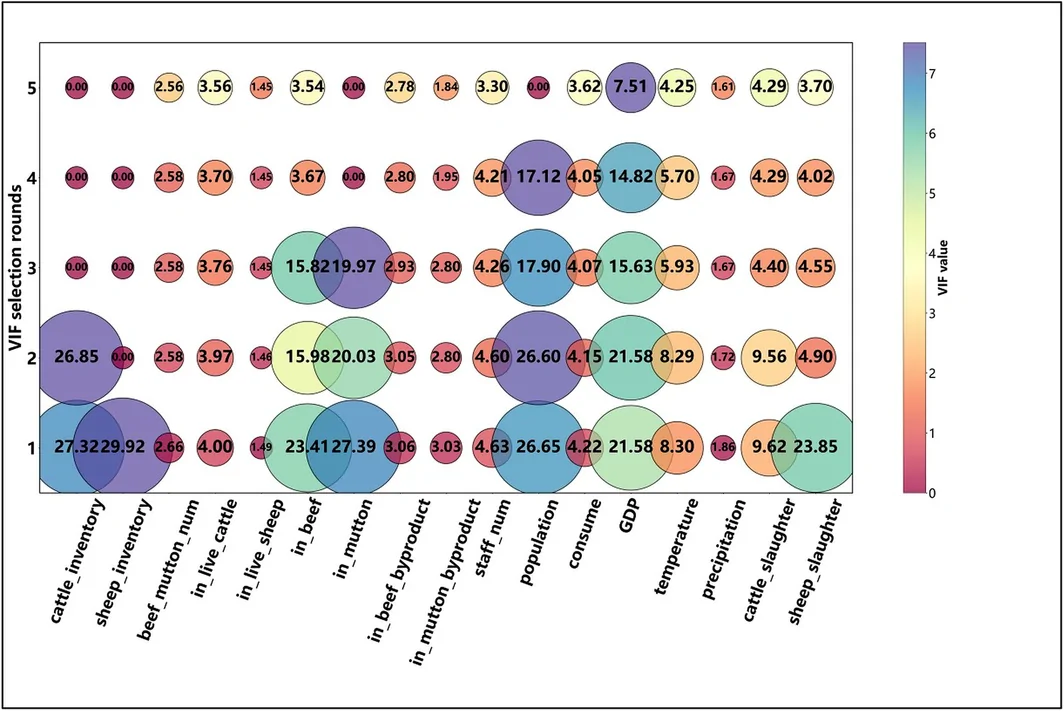
Results of VIF multi‐collinearity analysis.

**FIGURE 13:**
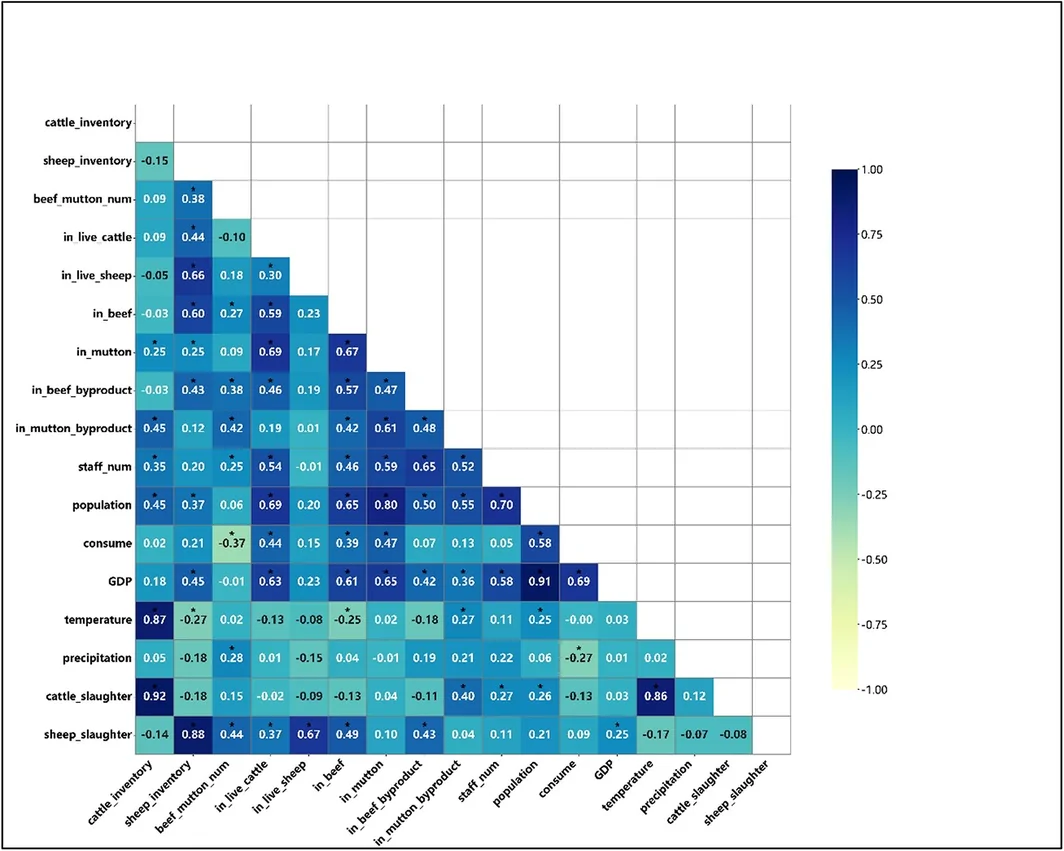
Spearman's correlation coefficient matrix (*indicates *p* value < 0.05).

Multi‐collinearity was systematically identified and mitigated using the variance inflation factor (VIF) during Bayesian spatiotemporal model construction. Iterative VIF analysis (Figure 12) initially revealed severe multi‐collinearity (VIF > 10) among sheep inventory, mutton transfer, cattle inventory, resident population, sheep slaughter, beef transfer and GDP. A stepwise elimination strategy was implemented, sequentially removing variables with the highest VIF (sheep inventory, cattle inventory, mutton transfer, resident population) and recalculating VIF values after each exclusion. After five screening rounds, remaining variables showed no significant multi‐collinearity, qualifying as independent predictors for single/multifactor models. Spearman's correlation analysis (Figure 13) validated this approach, confirming high correlations (all p<0.05) between sheep inventory and sheep slaughter (ρ=0.97), cattle inventory and cattle slaughter (ρ=0.90), mutton transfer and beef transfer (ρ=0.94) and resident population and GDP (ρ=0.90), supporting the rationality of variable exclusion.

#### 3.3.2 Univariate Analysis

After multicollinearity assessment, Bayesian spatiotemporal Model C was used for univariate analysis of 13 influencing factors (Table 4). Results showed three factors positively associated with brucellosis relative risk (RR): live sheep transfer (RR = 1.2050, 95% CI: 1.0241, 1.4269), per capita living consumption expenditure (RR = 1.2523, 95% CI: 1.0613, 1.4760) and sheep slaughter (RR = 1.7848, 95% CI: 1.2734, 2.6657). Ten other factors (beef/mutton yield, live cattle transfer, beef transfer, beef/mutton by‐product transfer, staff number, GDP, average temperature/rainfall, cattle slaughter) had no significant impact, with their RR 95% CIs including 1.

**TABLE 4:** RR and 95% confidence interval for the univariate Bayesian spatiotemporal model with respect to the different factor variables.

Factors	RR	95% CI
Beef and mutton yield	0.9681	(0.8044, 1.1693)
Transferred live cattle	1.0522	(0.8065, 1.3780)
Transferred live sheep	1.2050	(1.0241, 1.4269)
Transferred beef	0.8929	(0.6799, 1.1994)
Transferred beef by‐products	0.8346	(0.6853, 1.0187)
Transferred mutton by‐products	0.9947	(0.8465, 1.1700)
Number of staff	0.8886	(0.7225, 1.0940)
Per capita consumption expenditure	1.2523	(1.0613, 1.4760)
GDP	1.1086	(0.7787, 1.7294)
Average temperature	1.3603	(0.9918, 1.8853)
Average precipitation	0.9426	(0.8203, 1.0835)
Cattle slaughter	1.3143	(0.7750, 2.6703)
Sheep slaughter	1.7848	(1.2734, 2.6657)

#### 3.3.3 Multifactorial Analysis

Following VIF and univariate analyses, a multi‐factor Bayesian spatiotemporal model was constructed incorporating three key factors: live sheep transfer, per capita living consumption expenditure and sheep slaughter, to examine their impact on brucellosis incidence. As presented in Table 5, multivariate analysis results indicated that per capita consumption expenditure had no significant effect on brucellosis RR. However, live sheep transfer and sheep slaughtered exhibited positive correlations with brucellosis RR, with respective values of 1.1816 (95% CI: 1.0096, 1.3880) and 1.6175 (95% CI: 1.1883, 2.3546). Specifically, a 10% increase in live sheep transfer or sheep slaughter (relative to the baseline) was associated with an 18.16% or 61.75% rise in brucellosis RR, respectively. For interpretability in original units: all covariates were standardized (mean = 0, SD = 1) prior to model fitting. One SD increase in live sheep transfer corresponds to approximately 200,000 additional head transferred per year, associated with RR = 1.1816 (95% CI: 1.0096–1.3880). One SD increase in sheep slaughter corresponds to approximately 150,000 additional head slaughtered per year, associated with RR = 1.6175 (95% CI: 1.1883–2.3546). These SD values and original‐unit interpretations are provided in Table 5.

**TABLE 5:** RR and 95% confidence interval from the multivariate Bayesian spatiotemporal model, with original unit interpretation.

Factors	Original unit	One‐SD increment (original unit)	RR	95% CI
Transferred live sheep	10,000 head/year	20	1.1816	(1.0096, 1.3880)
Per capita consumption expenditure	Yuan/person/year	—	0.9632	(0.6370, 1.3302)
Sheep slaughter	10,000 head/year	15	1.6175	(1.1883, 2.3546)

## 4 Discussion

In our analysis of human brucellosis in Zhejiang Province from 2018 to 2023, a clear temporal trend is evident: the RR initially increased and then declined, with 2021 representing a significant inflection point. Before 2021, the risk remained relatively low (RR < 1), but post‐2021, it escalated into the high‐risk category. Although there was a slight decrease in 2023 compared to 2022, the risk remained elevated, suggesting a continued high‐risk situation in the short term. Spatially, the distribution of brucellosis risk in Zhejiang Province displays a discernible geographic pattern characterized by substantial spatial clustering. Particularly high‐risk hotspots are concentrated in the northern and southwestern regions, while the eastern coastal area exhibits a relatively lower risk. This spatial variation underscores differences in disease transmission across various geographic areas and highlights potential challenges and opportunities for regional prevention and control efforts. Our comprehensive examination of both spatial and temporal dimensions reveals the complex and varied evolution of brucellosis in Zhejiang Province. It should be noted that this is an ecological study conducted at the prefecture‐city aggregate level. The associations identified between covariates and brucellosis incidence are ecological associations and should not be interpreted as individual‐level causal relationships (ecological fallacy). These findings are hypothesis‐generating and individual‐level cohort studies (e.g., among slaughterhouse workers and live sheep transporters) are needed to confirm causal pathways. Distinct risk trends are observed across different regions, with high‐risk areas witnessing continuous escalation while low‐risk areas undergo fluctuating adjustments. These diverse municipal trends underscore the need for tailored prevention and control strategies to address the complex and dynamic nature of brucellosis in Zhejiang. This coexistence underscores the complexity and multi‐dimensional nature of the disease transmission mechanism.

In epidemiological studies, incidence and morbidity statistics serve as pivotal indicators for evaluating disease dissemination and regional risk differential [30]. Nevertheless, the intricate and dynamic nature of disease transmission is influenced by a multitude of factors, thereby necessitating a comprehensive risk assessment approach beyond single‐factor analyses. For instance, the classification of Jiaxing City in 2018 and Lishui City in 2023 varied based on assessments of morbidity versus RR. Similarly, Quzhou and Lishui Cities showed contrasting risk conclusions under different criteria, underscoring the limitations of singular indicator evaluations. Morbidity calculations primarily center on disease occurrence among a region's resident populace, often overlooking pivotal variables such as livestock populations and inter‐regional animal/product movements, which substantially impact disease propagation dynamics. Consequently, this study incorporates these variables into the risk assessment framework alongside traditional morbidity indicators, providing a more precise and holistic portrayal of disease transmission risk. Furthermore, tailored prevention and control strategies are proposed based on differentiated and precise risk categorizations for respective regions. High‐risk areas necessitate rigorous management and control interventions to mitigate disease dissemination, while medium‐risk areas require heightened surveillance to promptly trigger specific prevention and control protocols upon disease identification, thus averting progression to high‐risk status. Although low‐risk areas currently appear stable, maintaining routine prevention and management measures is crucial to preempt disease occurrence. This hierarchical approach ensures efficient resource mobilisation and maximizes prevention and control effectiveness.

Previous studies have identified ‘annual sheep slaughter’ practice as the primary contributor to human brucellosis incidence in Zhejiang Province from 2010 to 2018 (Huang et al. 2024). In the present study, we further interpret this finding in the context of Brucella species diversity. Available surveillance data from Zhejiang indicate that *B. melitensis* is the dominant strain circulating in sheep, which is consistent with the key role of sheep in driving human brucellosis transmission in major sheep‐rearing regions of China. The non‐significant effects of cattle‐related variables in our model are consistent with the generally lower zoonotic transmission risk associated with *B. abortus* . Importantly, individual‐level Brucella species typing data for human cases were not available in this study, which limits species‐specific source attribution and represents a notable limitation. Goat farming is negligible in Zhejiang and human cases due to *B. suis* are extremely rare. We also acknowledge that consumption of unpasteurized dairy products represents an important transmission route in brucellosis‐endemic regions. Although most commercially available dairy products in Zhejiang are pasteurized, infection risks from artisanal raw milk or dairy products from informal supply chains cannot be fully excluded. Future case investigations should incorporate detailed dietary exposure histories to further evaluate this potential transmission pathway. However, our study reveals a more intricate landscape where this traditional factor retains significance, yet the ‘importation of live sheep from outside the province’ emerges as another crucial determinant. Both factors significantly correlate with the RR of brucellosis, while other variables contribute minimally. Particularly noteworthy are Huzhou and Jiaxing Cities, which display notably elevated RRs of brucellosis. Huzhou, with 45% of the province's sheep production and Jiaxing, both situated on Zhejiang's border and serving as major hubs for inter‐provincial live sheep transfer, face considerable challenges in brucellosis prevention and control due to the intricate and frequent sheep circulation within their jurisdictions. Illegal transportation practices, regulatory enforcement difficulties and the relatively low costs associated with violations exacerbate disease dissemination in these urban centers, thereby corroborating previous research findings (Liu et al. 2019; Wu et al. 2020). To effectively mitigate this, we propose strategic recommendations: Firstly, enhancing oversight over sheep slaughter practices and inter‐provincial live sheep transfers is paramount, necessitating the implementation of stringent regulatory mechanisms to establish closed‐loop management systems that significantly reduce the risk of disease transmission. Secondly, recognizing the complex and multifaceted nature of brucellosis transmission, we recommend the integration of multiple influencing factors into regional risk assessments. Additionally, promoting cross‐regional and cross‐sectoral collaboration mechanisms is essential for fostering synergistic approaches to brucellosis prevention and control.

This study serves as a valuable scientific resource for brucellosis prevention and control in Zhejiang. However, it is essential to acknowledge certain limitations, such as the coarse temporal resolution (annual) and the constraints imposed by the dataset (e.g., absence of monitoring data for transferred cattle and sheep). (1) Annual temporal resolution may obscure finer seasonal dynamics. (2) Animal‐side data (livestock seroprevalence) were not available at the prefecture‐city level. (3) As an ecological study, associations are group‐level and subject to the ecological fallacy; results may be sensitive to the modifiable areal unit problem (MAUP). (4) Brucella species typing data for human cases were unavailable, limiting reservoir‐specific attribution. (5) Animal‐side data (livestock seroprevalence) were not available at the prefecture‐city level. (6) Potential covariates including land use and dietary exposures (e.g., raw dairy) were not incorporated. (7) Municipal‐level aggregation may mask sub‐city heterogeneity. To address these limitations effectively, we propose the establishment of a comprehensive production monitoring and epidemic surveillance system throughout Zhejiang. This system would enable real‐time data collection, integration and sharing, thereby enhancing the ability to accurately identify brucellosis epidemiological trends and optimise prevention and control strategies. Future research should prioritise incorporating animal seroprevalence as key covariates, fully consider the policy characteristics of Class‐II endemic areas and integrate animal health surveillance, individual‐level epidemiological data and real‐time livestock movement tracking to build a comprehensive One Health monitoring system for brucellosis prevention in Zhejiang.

## Funding

This work was supported by the Zhejiang Province Three Rural Nine‐party Science and Technology Cooperation Project (2024SNJF043), National Natural Science Foundation of China (12201562), Open Project Funded by the Ministry of Education Key Laboratory of NSLSCS (202503), Science Foundation of Zhejiang Sci‐Tech University (22032171‐Y).

## Ethics Statement

The authors state that ethical approval was not required as this study used the data from official platforms and statistical data.

## Conflicts of Interest

The authors declare no conflicts of interest.

## Supporting information


**Figure S1:** Year‐by‐year temporal evolution of the posterior probability *P*(*RR* > 1) across Zhejiang's municipalities (2018–2023).
**Figure S2:** Spatial distribution of total reported cases of human brucellosis in each region in Zhejiang (2018–2023).

## Data Availability

All relevant data used in this study are detailed in the manuscript (see Subsection 2.1 ‘Data Sources’) and are available from the corresponding author upon reasonable request.
