# Supplementary material for: Spatiotemporal Analysis of the Primary Factors and Risk Quantification of Human Brucellosis Prevention and Control in Zhejiang Province of China
Source: Zoonoses Public Health. 2026 Jul 22;73(6):547–62. doi: 10.1111/zph.70076 (PMC13535741; doi:10.1111/zph.70076)
Supplement: Supplementary file 1 [file ZPH-73-547-s001.docx]

**Supplementary Materials**

**Fig. S1** Year-by-year temporal evolution of the posterior probability $P(RR>1)$ across Zhejiang's municipalities (2018–2023).

**Fig. S2** Spatial distribution of total reported cases of human brucellosis in each region in Zhejiang

(2018-2023).
